# Protective Role of Key Micronutrients in Chemotherapy-Induced Organ Toxicity: A Comprehensive Review of Mechanistic Insights and Clinical Implications

**DOI:** 10.3390/nu17172838

**Published:** 2025-08-31

**Authors:** Ioannis Konstantinidis, Sophia Tsokkou, Eleni Gavriilaki, Georgios Delis, Theodora Papamitsou

**Affiliations:** 1 Department of Medicine, Faculty of Health Sciences, Aristotle University of Thessaloniki, 54124 Thessaloniki, Greece; stsokkou@auth.gr; 2 2nd Propedeutic Department of Internal Medicine, Haematology Unit-Haemophilia Centre of Northern Greece, Hippokration General Hospital of Thessaloniki, Aristotle University of Thessaloniki, 54124 Thessaloniki, Greece; gavriiel@auth.gr; 3 Laboratory of Pharmacology, School of Veterinary Medicine, Faculty of Health Sciences, Aristotle University of Thessaloniki, 54124 Thessaloniki, Greece; delis@vet.auth.gr; 4 Laboratory of Histology-Embryology, Department of Medicine, Faculty of Health Sciences, Aristotle University of Thessaloniki, 54124 Thessaloniki, Greece

**Keywords:** chemotherapy-induced toxicity, cardiotoxicity, hepatotoxicity, nephrotoxicity, magnesium, selenium, zinc, metallothionein, vitamin D, oxidative stress, protective micronutrients, minerals, trace elements

## Abstract

Background/Objectives: Systemic toxicities to key organs like the heart, liver, and kidneys impair the efficacy of chemotherapy in cancer treatment. These toxicities are caused by oxidative stress, inflammation, mitochondrial malfunction and ferroptosis, causing clinical morbidity and possibly impaired adherence to treatment. This review, also, examines how magnesium, selenium, zinc and vitamin D protect against chemotherapy-induced cardiotoxicity, hepatotoxicity and nephrotoxicity. Methodology: A complete literature search of PubMed (MEDLINE), Scopus, Cochrane Library and Embase was used to synthesize data till 29 June 2025. Studies included randomized and non-randomized trials, cohort studies, case series (≥3 patients), and relevant systematic reviews. To contextualize pathways, preclinical in vivo and in vitro studies were studied independently. Patients undergoing systemic chemotherapy and magnesium, selenium, zinc or vitamin D therapies were eligible. Supplementation’s safety and organ-specific toxicity were investigated. Results: Magnesium protected against cisplatin-induced nephrotoxicity via modulating renal transporters and oxidative defenses across chemotherapy regimens. Selenium supplementation has strong antioxidant and anti-inflammatory characteristics, especially in avoiding cardiac and hepatic injury, although its nephroprotective potential was formulation-dependent. Zinc’s activity was connected to metallothionein-mediated redox stabilization, inflammatory regulation, and cardiac and hepatic resilience. Vitamin D and its analogs reduced cardiotoxicity and nephrotoxicity through mitochondrial preservation and immunomodulatory signaling. Conclusions: To date, magnesium, selenium, zinc, and vitamin D have been shown to reduce chemotherapy-related organ toxicities. Preclinical studies are promising, but randomized clinical trials are needed to prove therapeutic effectiveness and oncologic safety.

## 1. Introduction

Chemotherapy is fundamental in the treatment of oncological patients, offering a systemic control over malignant cells. Despite its effectiveness, chemotherapy is known for its broad-spectrum toxicity, affecting both cancerous and healthy tissues. As the chemotherapeutic agents are metabolized and excreted mostly by the liver or kidneys, they are very prone to show toxic effects towards these organs. The adverse effects range from nephrotoxicity, hepatotoxicity, neurotoxicity and hematological suppression to gastrointestinal distress that can significantly impair patients’ quality of life and compromise treatment adherence [1]. The fundamental mechanisms of chemotherapy-induced toxicity often encompass oxidative stress, inflammation, mitochondrial dysfunction and ferroptosis [2].

Chemotherapeutic regimens including cisplatin (or other platinum compounds), doxorubicin (or other anthracyclines) and methotrexate produce excessive reactive oxygen species (ROS) and as a result, surpass the capacity of cellular antioxidant defense mechanisms. This imbalance leads to lipid peroxidation, DNA damage and protein oxidation, which contribute to cytotoxicity in both malignant and healthy cells. The deregulation of redox-sensitive pathways, such as NF-κB and Nrf2/HO-1, intensifies tissue damage, especially in neurological tissues [1,3].

Inflammation can be caused by chemotherapy and affects its efficacy, as well. Because chemotherapy has the ability of stimulating and activating the innate immune responses, it can result in the secretion of pro-inflammatory cytokines, such as TNF-α, IL-6, and IL-1β [4]. The inflammatory cascade is frequently governed by the NF-κB pathway and inflammasome activation, which adds to acute toxicity and may facilitate tumors’ recurrence via tissue remodeling and immune suppression. Chronic inflammation has been associated with resistance to treatment and prolonged organ damage [4,5,6].

Furthermore, chemotherapy-driven mitochondrial dysfunction could lead to bioenergetic alterations, increased oxidative stress and disruptions in calcium signaling, ultimately affecting cellular processes and potentially causing chemotherapy-induced peripheral neuropathies, cognitive impairment and muscle weakness [7]. Pharmaceutical regimens, like paclitaxel and vincristine, influence mitochondrial membrane potential (ΔΨm), induce calcium (Ca^2+^) dysregulation, and obstruct ATP production, leading to neuronal excitability and peripheral neuropathy. Platinum-based agents, such as cisplatin and oxaliplatin, form adducts with mitochondrial DNA, hindering replication and transcription, which ultimately results in energy depletion and apoptotic cell death [8,9,10].

Ferroptosis is a controlled, iron-dependent form of cell death characterized by lipid peroxidation and glutathione depletion. Chemotherapeutic agents, including cisplatin and doxorubicin, have shown the ability to induce ferroptosis by disrupting the GPX4–GSH–SLC7A11 axis and facilitating ferritinophagy. While ferroptosis may contribute to tumors’ suppression, its activation in healthy tissues, particularly in the kidneys, liver and neurons, can exacerbate organ damage. Recent studies suggest that natural compounds, like ginger and 6-gingerol, may mitigate damage linked to ferroptosis [11,12].

Common chemotherapeutic agents include anthracyclines, like doxorubicin, that are known to cause cardiotoxicity. Specifically, a study reported that anthracyclines can lead to symptomatic heart failure in 2–4%, asymptomatic decrease in LVEF in 9–11%, arrhythmia in 12% or so and a rise in cardiac biomarkers in 30–35% of treated patients [13]. Alkylating agents, such as cyclophosphamide, ifosfamide, cisplatin, carmustine, busulfan, chlormethine and mitomycin, are also associated with cardiotoxicity. Additionally, chemotherapeutic agents that may induce a cardiac treatment adverse event include paclitaxel, etoposide, teniposide, the vinca alkaloids, fluorouracil, cytarabine, amsacrine, cladribine, asparaginase, tretinoin and pentostatin [14].

Cisplatin is a cytotoxic drug that triggers the development of many toxicities, with the most common being nephrotoxicity and ototoxicity. A recent prospective study found that 44% of patients treated with cisplatin-based chemotherapy developed toxicity. Specifically, 15% of patients experienced nephrotoxicity, 9% ototoxicity and 27% gastrointestinal toxicity [15].

When it comes to hepatotoxicity, classes of chemotherapeutic agents causing hepatotoxicity include antitumor antibiotics, alkylating agents, platinum agents, antimetabolites, antimicrotubular agents and topoisomerase inhibitors [16].

### Objectives

The objective of this review is the identification and evaluation of the protective roles of minerals, trace elements and vitamins in mitigating the toxic effects associated with cancer chemotherapy. Furthermore, this review will focus on the protective role of selenium, zinc, magnesium and vitamin D in mitigating chemotherapy-induced cardiotoxicity, hepatotoxicity and nephrotoxicity.

## 2. Materials and Methods

The evidence base on micronutrients’ protection against chemotherapy-related toxicities is markedly heterogeneous. The variability spans cancer types and regimens, such as anthracyclines, platinum agents and antimetabolites; populations (age, comorbidity, baseline deficiencies); interventions (magnesium, selenium, zinc, vitamin D); comparators (placebo, standard care, alternative supplements); and outcome definitions (clinical events, organ-specific biomarkers, imaging). Randomized trials are limited for several micronutrients and indications, while much of the literature comprises small cohorts, case series, and preclinical studies. Under these conditions, a traditional meta-analysis risks misleading pooled estimates and loss of clinically salient evidence. We therefore prespecified a narrative synthesis to (i) capture the full breadth of clinical and preclinical evidence without excluding informative smaller studies; (ii) map diagnostic practices and outcome measures across organ systems; (iii) contrast prophylactic versus therapeutic strategies and routes of administration; and (iv) identify methodological gaps to guide future prospective studies.

A comprehensive literature search was performed to identify studies reporting systemic toxicity induced by chemotherapeutic regimens and the protective role of vitamins, minerals and trace elements. PubMed (MEDLINE), Scopus, Cochrane Library and Embase electronic databases were queried from inception to 29 June 2025. Search terms combined controlled vocabulary and free-text words and included the following keywords: “chemotherapy toxicity”, “cardiotoxicity”, “neurotoxicity”, “nephrotoxicity”, “pulmonary toxicity”, “gastrointestinal toxicity”, “hepatotoxicity”, “minerals”, “vitamins”, “trace elements”, “protective agents”, “selenium”, “zinc”, “magnesium”, “vitamin D” and “supportive care”. References were reviewed for the identification of any additional relevant articles.

This review was restricted to full-text studies published in peer-reviewed journals in the English language. Eligible studies had to meet the following criteria, outlined utilizing the Population, Interventions, Comparators and Outcomes (PICO) framework:

Population: Humans receiving systemic chemotherapy for any malignancy. Preclinical in vivo studies modeling chemotherapy-induced organ toxicity were included to contextualize mechanisms and dosing, with findings synthesized separately from clinical evidence. In vitro-only studies were excluded unless directly linked to included in vivo/clinical data.

Interventions: Minerals, trace elements or vitamins administered as single agents or defined combinations (with an a priori focus on magnesium, selenium, zinc, and vitamin D), delivered by any route (oral, enteral, intravenous) for prophylaxis or treatment of toxicity.

Comparators: Placebo, no supplementation, standard care or alternative micronutrient regimens.

Outcomes: Organ-specific toxicity outcomes, such as cardiotoxicity, neurotoxicity, nephrotoxicity, pulmonary toxicity, gastrointestinal toxicity, hepatotoxicity, safety/adverse events related to supplementation and, when reported, oncologic outcomes to assess any signal of tumor protection or treatment interference.

Study designs: Randomized and non-randomized interventional studies, prospective and retrospective cohorts, case–control studies, case series (≥3 patients) and relevant systematic reviews and meta-analyses (used to identify primary studies and, when appropriate, summarized narratively). Single-patient case reports were included only when detailing rare toxicities or interventions otherwise under-represented. Duplicate records, review articles, protocols and guidelines, animal studies, conference abstracts and presentations, preprints, clinical trials under patient recruitment or without published results, ongoing clinical trials and studies deemed irrelevant were excluded.

To ensure accuracy and objectivity, two independent reviewers (I.K. and S.T.) initially screened the titles and abstracts in a double-blinded process. For studies that passed this initial screening, the full texts were obtained and further evaluated to determine their final eligibility. Any discrepancies during the screening process were resolved by a third reviewer (T.P.). Duplicates were removed prior to screening. All relevant data were extracted regarding the type of toxicity, chemotherapeutic agents involved, protective compounds used, mechanisms of action and clinical outcomes. The PRISMA flow diagram (Supplementary Figure S1) outlines the review selection and exclusion process.

## 3. Results

### 3.1. Chemotherapy-Induced Toxicity per System

Cardiotoxicity from cancer therapies spans arrhythmias, ischemia, hypertension and ventricular dysfunction. It is delineated by a reduced left ventricular ejection fraction (LVEF) or abnormal septal motion, clinical heart failure, tachycardia, or LVEF falling below 55% with heart failure signs after treatment. Two patterns are recognized: type I (classically anthracycline-related), dose-dependent and often irreversible, with myocyte loss, and type II (classically trastuzumab-related), typically non–dose-dependent and potentially reversible, though this dichotomy is imperfect. Incidence ranges from acute presentations in under 1% to chronic dysfunction in 1.6 up to 5% one year or more after therapy, and partial reversibility has been observed with heart failure therapy even after anthracyclines, while irreversible scarring has been reported with trastuzumab [17,18,19,20,21,22].

Neurotoxicity is a major dose-limiting complication after myelosuppression, affecting the peripheral and central nervous systems despite protective barriers. Peripheral neuropathy with sensory symptoms predominates, while central events include seizures, encephalopathy, delirium and cerebellar dysfunction [23,24]. Classic culprits are vincristine, methotrexate, cisplatin and cytarabine, with newer agents such as brentuximab and blinatumomab also implicated [23,25].

Nephrotoxicity reflects the kidney’s central role in drug elimination and encompasses AKI, CKD, proteinuria, electrolyte derangements and thrombotic microangiopathy. Risk rises with hypovolemia, concomitant nephrotoxins, contrast media and baseline renal disease. Cisplatin, methotrexate and gemcitabine are prototypic offenders, while EGFR inhibitors and immune checkpoint inhibitors contribute to modern regimens. Early risk stratification, close monitoring, dose adjustment and preventive hydration are more than essential [26,27,28,29,30,31].

Pulmonary toxicity is common, driven by direct cytotoxic and immune-mediated injury and compounded by infection in immunosuppressed patients. Beyond cardiotoxicity, anthracyclines have been associated with clinically significant lung injury, including fibrosis. Nonspecific symptoms (fever, cough, dyspnea) complicate diagnosis. High-resolution CT is preferred, revealing interstitial infiltrates, diffuse alveolar damage, nonspecific interstitial pneumonia, eosinophilic pneumonia, organizing pneumonia, hemorrhage and capillary leak patterns [32,33].

Gastrointestinal toxicity and, particularly, diarrhea and mucositis result from damage to the rapidly developing epithelium, leading to barrier disruption, inflammation and dysmotility. Diarrhea impacts as many as 80% of patients, and mucositis occurs by a five-phase cascade influenced by ROS, DNA damage, cytokines and stem cell apoptosis, exacerbated by chemotherapy-induced dysbiosis and enteric nervous system contributions through oxidative regulation of ion channels and neuronal excitability. Irinotecan, 5-fluorouracil, cytarabine, cisplatin and taxanes are frequently involved. Conventional therapies mostly provide palliative care, whereas novel strategies (amifostine, CXCL9, GLP-1/2, GPER agonists, cystine/theanine) seek to mitigate oxidative and inflammatory factors and maintain epithelial integrity [34].

Hepatotoxicity is frequently idiosyncratic, not dose-dependent or predictable, and it varies from moderate transaminase increases to steatohepatitis, sinusoidal obstruction syndrome and severe liver failure. The agents comprise methotrexate, cyclophosphamide, oxaliplatin and irinotecan. The mechanisms focus on oxidative damage, mitochondrial failure and immune-mediated inflammation. Pre-existing liver illness or viral hepatitis increases the risk. Management depends on baseline and serial surveillance, precise distinction of DILI from disease progression, targeted imaging or biopsy and customization of regimens, with dosage modifications according to hepatic function [35,36,37,38,39,40].

**Figure 1:**
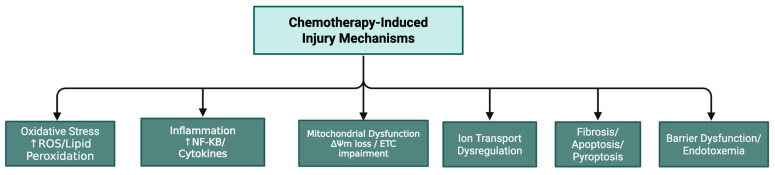
Schematic overview of chemotherapy-induced injury pathways. Chemotherapy initiates multiple damaging processes, including oxidative stress with accumulation of ROS and lipid peroxidation, inflammatory signaling via NF-κB and cytokines, mitochondrial dysfunction with ΔΨm loss and ETC impairment, ion transport dysregulation, barrier disruption leading to endotoxemia and cell death through fibrosis, apoptosis and pyroptosis. Note: ↑: increased.

All evidence regarding chemotherapy-induced organ-specific toxicity is provided with details in Table 1 and depicted in Figure 1.

### 3.2. The Role of Micronutrients in Chemotherapy-Induced Toxicity

In contrast to macronutrients, like carbohydrates, proteins and fats, where a significant quantity is required, micronutrients are required in trace amounts [41]. In the family of micronutrients vitamins and minerals are included, demonstrating a pivotal role in multiple physiological processes [41]. Micronutrients are essential for good health and are involved in many processes, like immune function, bone health, blood clotting, nerve function and many more [41]. They are essential cofactors in enzymatic reactions, cellular defense mechanisms and tissue repair. Their supplementation during chemotherapy has shown promise in reducing organ-specific toxicities [42]. Table 2 presents a short summary of toxicities, the protective agents, the mechanism of action and any additional comments present.

Several protective agents have shown promising results in reducing chemotherapy-induced toxicity and alleviating the adverse effects. Cardiotoxicity, for instance caused by anthracyclines, can be reduced using agents like dexrazoxane, which chelates iron and lowers ROS species, in combination with Coenzyme Q10, selenium, vitamin E, and omega-3 fatty acids that support mitochondrial function and provide antioxidant and anti-inflammatory benefits. Dexrazoxane is FDA-approved for anthracycline-induced cardiotoxicity [43]. When it comes to the neurotoxicity caused by chemotherapy treatment options common with agents like oxaliplatin and vincristine, they may be alleviated by magnesium, calcium, vitamins B6 and B12, alpha-lipoic acid and glutamine, which stabilize neuronal membranes, support myelin synthesis and reduce excitotoxicity. For nephrotoxicity, especially with cisplatin and methotrexate, hydration, magnesium, N-acetylcysteine and selenium are critical, as they dilute nephrotoxins, prevent electrolyte imbalance and reduce oxidative damage to renal tubules [44]. Pulmonary toxicity, like drug-induced pneumonitis, may be reduced with N-acetylcysteine, corticosteroids, vitamins C and E and curcumin, which act as antioxidants, reduce inflammation and modulate cytokine responses [45]. In gastrointestinal toxicity cases, agents like L-glutamine, cystine/theanine, vitamin D and probiotics help repair mucosal damage, reduce apoptosis and restore gut microbiota balance, showing promise in managing mucositis and chemotherapy-induced diarrhea [46]. Finally, hepatotoxicity is usually observed with methotrexate, irinotecan and platinum agents’ administration and can be countered with silymarin, N-acetylcysteine, vitamin E and ursodeoxycholic acid, which enhance glutathione synthesis, reduce lipid peroxidation and improve bile flow [47].

### 3.3. Minerals and Trace Elements in Cardiac Injury

Minerals demonstrate a crucial role in sustaining cardiac electrophysiology, vascular tone and myocardial integrity, rendering them essential in the setting of chemotherapy-induced cardiotoxicity [48]. Crucial electrolytes, including potassium, calcium, magnesium and sodium, are vital for the optimal operation of heart cells. Potassium and sodium govern membrane potentials and the propagation of action potentials, but calcium is pivotal in excitation–contraction coupling [49]. Magnesium, functioning as a physiological calcium antagonist, aids in stabilizing heart rhythm and preventing arrhythmias [50]. Chemotherapeutic medicines, especially anthracyclines and platinum-based medications, can disturb the homeostasis of these minerals, resulting in heightened vulnerability to arrhythmias, diminished contractility and fluid imbalance. Alongside these primary minerals, trace elements, although needed in lesser amounts, are similarly crucial for cardiovascular health and protection against chemotherapy-induced harm [51,52].

Zinc and selenium are also crucial components of antioxidant enzymes such as superoxide dismutase and glutathione peroxidase, which mitigate oxidative stress generated by chemotherapeutic agents. Copper contributes to mitochondrial respiration and antioxidant defense, while iron, although essential for oxygen transport and cellular metabolism, can exacerbate oxidative injury when present in excess, particularly through the Fenton reaction [53,54]. Dysregulation of these trace elements can compromise myocardial repair mechanisms and amplify the cardiotoxic effects of chemotherapy. Therefore, maintaining optimal levels of both minerals and trace elements is increasingly recognized as a key strategy in preserving cardiac function and minimizing adverse outcomes during cancer treatment [55].

In the following section, this review will focus on the protective role of selenium, zinc, magnesium and vitamin D in mitigating chemotherapy-induced cardiotoxicity, hepatotoxicity and nephrotoxicity.

## 4. Discussion

### 4.1. Magnesium

#### 4.1.1. Protective Role of Magnesium in Chemotherapy-Induced Nephrotoxicity

Magnesium demonstrates a clinically and mechanistically substantiated renoprotective effect in cisplatin-induced nephrotoxicity, with adult cohort data showing that prophylactic intravenous magnesium (typically 2 g on the day of chemotherapy) reduces the incidence of acute kidney injury (AKI) or mortality (OR 0.80; 95% CI 0.66–0.97), particularly in patients < 65 years, women, diabetics, those with baseline eGFR ≥ 90 mL/min/1.73 m^2^ and higher baseline magnesium levels (2.0–2.2 mg/dL) [56]. Meta-analysis of 11 retrospective studies (n = 1688) further supports this benefit (pooled OR 0.22; 95% CI 0.14–0.35), most pronounced at cisplatin doses ≤ 50 mg/m^2^, yet present across dosing ranges [57]. Mechanistically, repletion with magnesium counteracts cisplatin-induced proximal tubular injury by normalizing hypomagnesemia-driven upregulation of basolateral OCT2 and downregulation of apical MATE1, thereby limiting accumulation of intracellular platinum; it also stabilizes tubular membranes, supports Na^+^/K^+^-ATPase, modulates calcium channels and suppresses oxidative and inflammatory cascades [56,57,58]. These clinical and mechanistic signals suggest potential for dose- or baseline magnesium-guided supplementation strategies, although optimal regimens remain undefined. In contrast, a randomized pediatric trial in carboplatin-treated patients found no biomarker or functional renal benefit from short-course oral magnesium oxide (250 mg/day for two weeks), likely reflecting the lesser nephrotoxicity of carboplatin, formulation and dose differences, brief pre-chemotherapy exposure and small sample size [58]. Thus, while the pediatric carboplatin data caution against uncritical extrapolation, the biological rationale and consistent adult cisplatin evidence provide a robust case for targeted magnesium prophylaxis.

#### 4.1.2. Protective Role of Magnesium in Chemotherapy-Induced Hepatotoxicity

Across preclinical models involving distinct anticancer agents, magnesium isoglycyrrhizinate (MgIG) consistently mitigates chemotherapy-induced hepatotoxicity by targeting mitochondrial dysfunction, oxidative stress and innate immune activation across diverse toxicants, including alectinib, arsenic trioxide (ATO) and methotrexate (MTX), with suppression of COX-2-centered inflammation and modulation of the gut–liver axis [59,60,61,62]. In alectinib injury, MgIG restored mitochondrial membrane potential and electron transport chain function, reduced ROS, normalized Nrf2/HO-1 and inhibited NF-κB-linked NLRP3 inflammasome pyroptosis, with N-acetyl-L-cysteine recapitulating benefits [59]. In ATO models, it lowered ROS and lipid peroxidation, replenished glutathione and antioxidant enzymes, reduced IL-1β, IL-6, TNF-α and shifted Bax/Bcl-2 towards survival via Keap1-Nrf2 modulation [61]. In MTX hepatotoxicity, MgIG reinforced intestinal barrier proteins (ZO-1, claudin-1, E-cadherin), decreased permeability and LPS–TLR4 signaling, remodeled microbiota (restoring Lactobacillus, SCFAs), promoted anti-inflammatory M2 macrophage polarization and reduced oxidative stress, apoptosis, fibrosis, and COX-2 expression, with Lactobacillus supplementation or fecal microbiota transplantation reproducing protection [60,62]. These converging mitochondrial- and gut-centered mechanisms preserved hepatocellular architecture and function, suggesting that MgIG acts on proximal pathogenic nodes to avert divergent cell-death pathways, and that translational development should focus on mechanistic biomarkers, dose optimization, pharmacokinetics and oncologic safety before clinical adoption [59,60,61,62].

#### 4.1.3. Protective Role of Magnesium in Chemotherapy-Induced Cardiotoxicity

In multiple cardiotoxicity models involving arsenic trioxide, doxorubicin and cyclosporine A, magnesium—administered as magnesium isoglycyrrhizinate (MgIG), magnesium sulfate or dietary magnesium—consistently mitigated myocardial injury by modulating oxidative stress, inflammation, mitochondrial apoptosis, calcium handling, electrophysiology and vascular–hemodynamic balance [63,64,65,66]. MgIG restored antioxidant defenses, including superoxide dismutase, catalase and glutathione peroxidase, and it activated Nrf2, thereby reducing lipid peroxidation and ROS, while downregulating TLR4/NF-κB-mediated cytokine amplification and lowering pro-inflammatory mediators [63,65]. Anti-apoptotic effects were evident through reduced Bax and caspase-3, increased Bcl-2 and decreased TUNEL positivity, indicating preserved mitochondrial integrity [63,65]. Magnesium sulfate ameliorated doxorubicin-induced QT prolongation, contractile dysfunction and glutathione depletion, likely via calcium antagonism at contractile proteins, modulation of L-type Ca^2+^ channels, sarcoplasmic reticular calcium pumps, Na^+^/Ca^2+^ exchange and Na^+^/K^+^-ATPase activity [64]. Dietary magnesium, particularly with potassium, attenuated cyclosporine A-induced vascular remodeling, fibrosis and hypertension, highlighting extracellular milieu as a determinant of cardioprotection [66]. Benefits were dose-responsive, accompanied by normalization of biomarkers (CK, CK-MB, LDH) and histological preservation, and they occurred without adverse effects when magnesium was administered alone, with some effects independent of serum magnesium changes, suggesting intracellular or compartment-specific actions [63,64,65,66].

**Figure 2:**
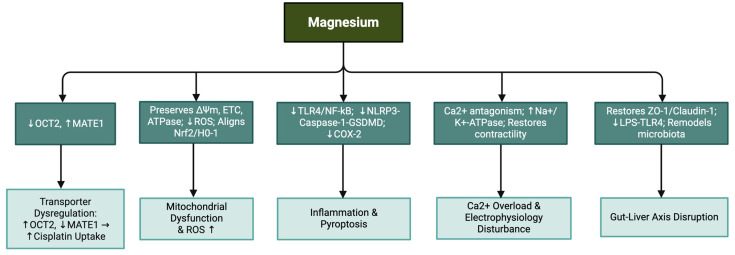
Proposed mechanisms of magnesium’s protective actions in mitigating chemotherapy-induced organ injury. Magnesium supplementation counteracts these effects through transporter regulation, mitochondrial preservation, anti-inflammatory signaling, calcium antagonism and maintenance of gut barrier integrity. Note: ↓: decreased; ↑: increased.

Proposed mechanisms of magnesium’s protective role in mitigating chemotherapy-induced organ injury are summarized in Table 3 and Figure 2.

### 4.2. Selenium

Selenium is an essential mineral naturally found in or added to many foods. It can also be consumed as a dietary supplement. It is a constituent of 25 selenoproteins, including thioredoxin reductases, glutathione peroxidases and selenoprotein P. Selenoproteins play a crucial role in thyroid hormone metabolism, DNA synthesis, reproduction and protection from oxidative damage and infection [67,68]. Selenium offers multi-organ protection via antioxidant, anti-inflammatory and mitochondrial-preserving effects but has a narrow therapeutic window, with a U-shaped dose–response. Benefits occur at low-to-moderate doses, but higher or prolonged exposures, especially with some nanoparticle formulations, may be harmful, requiring adherence to upper intake limits, baseline status checks and monitoring to ensure safety during chemotherapy [67,68].

#### 4.2.1. Protective Role of Selenium in Chemotherapy-Induced Hepatotoxicity

In multiple preclinical models of chemotherapy-induced hepatotoxicity across diverse agents, including doxorubicin, adriamycin, cyclophosphamide and cisplatin, selenium has consistently demonstrated hepatoprotective effects through restoration of redox balance, suppression of inflammatory pathways and attenuation of cell death, with correlated biochemical, histopathological and genomic benefits [69,70,71,72,73,74]. Selenium’s predominant mechanism involves reinforcement of antioxidant defenses via upregulation of selenoproteins such as glutathione peroxidase, superoxide dismutase and catalase, activation of Nrf2-dependent cytoprotective transcription, replenishment of glutathione and reduction in lipid peroxidation and nitric oxide, thereby mitigating oxidative and nitrosative stress [69,71,72,74]. These redox effects are complemented by inhibition of NF-κB signaling and pro-inflammatory cytokines (TNF-α, IL-1β, IL-6) [69,70], preservation of mitochondrial membrane potential and ATP synthesis [71] and modulation of apoptotic mediators, including Bax, Bcl-2 and caspase-3 [69,70,72]. Formulation, dosage and timing are critical, as low to moderate dosing confers protection, whereas excessive selenium can induce pro-oxidant damage [70]. Additional mechanisms include enhancement of conjugative detoxification pathways (UGTs) and mitigation of genotoxicity through reduced DNA strand breaks and chromosomal aberrations [72]. In cisplatin models, selenium also restored the GSH/GSSG ratio and may directly sequester reactive platinum intermediates without impeding antitumor efficacy [73]. Collectively, these findings outline a coherent mechanistic framework in which selenium, within a defined therapeutic window, exerts multifaceted hepatic protection by integrating antioxidant, anti-inflammatory, mitochondrial-preserving and anti-apoptotic actions [69,70,71,72,73,74].

#### 4.2.2. Protective Role of Selenium in Chemotherapy-Induced Cardiotoxicity

Selenium demonstrates cardioprotective properties against myocardial injury generated by anthracyclines and cyclophosphamide through a combination of antioxidant, anti-inflammatory and anti-apoptotic pathways, as evidenced by preclinical studies and limited clinical data [75,76,77,78,79,80]. Selenium replenishes glutathione, augments glutathione peroxidase activity, reinstates superoxide dismutase and mitigates lipid peroxidation products such malondialdehyde and 4-hydroxynonenal, thus restoring redox equilibrium [75,76,78,79]. The upregulation of Nrf2 and its target genes, along with the suppression of NF-κB-mediated cytokine production and decreases in TNF-α, IL-1β and IL-18, alleviates inflammatory amplification [75,76,78]. Selenium downregulates PARP1 and TRPM2, hence diminishing oxidative stress-induced calcium influx and mitochondrial apoptotic signaling, as demonstrated by decreased caspase-3/9 activity, reduced Bax levels, increased Bcl-2 levels and diminished cardiomyocyte loss [75,76,77]. Supplementary advantages encompass the maintenance of microvascular and sarcolemmal integrity, reduction of ischemia-modified albumin, alleviation of DNA double-strand breaks (γH2AX) and stabilization of contractile proteins [77,79]. Selenium-enriched diets have preserved the ejection fraction in murine doxorubicin models, whereas in juvenile patients, low selenium levels were associated with increased ProBNP and echocardiographic abnormalities, with supplementation enhancing biomarkers and function. These findings collectively outline a selenium-mediated cardioprotective network involving Nrf2 activation, NF-κB inhibition, modulation of TRPM2-associated calcium dysregulation and regulation of apoptotic checkpoints, resulting in maintained myocardial structure and function without adverse effects at effective doses.

#### 4.2.3. Protective Role of Selenium in Chemotherapy-Induced Nephrotoxicity

Selenium acts as a context-dependent modulator of key molecular pathways in chemotherapy-induced nephrotoxicity, with most consistent benefits in acute cisplatin or cyclophosphamide injury models and context-dependent variability under prolonged exposure [81,82,83,84,85,86]. In cisplatin and cyclophosphamide settings, selenium, administered as sodium selenite, nano-selenium or turmeric-selenium nanoparticles, restores redox balance by elevating glutathione peroxidase, catalase, superoxide dismutase and glutathione, while reducing malondialdehyde, advanced oxidation protein products and nitric oxide and activating the Nrf2–HO-1 axis [82,83,84,86]. These effects align with improved tubular morphology, partial normalization of creatinine and urea and, clinically, preserved GFR when combined with vitamin E [85]. Selenium concurrently suppresses NF-κB activity and pro-inflammatory cytokines (IL-1β, TNF-α, IL-6) [82,83], mitigates extracellular matrix remodeling by lowering MMP-9 [83] and shifts apoptotic signaling towards survival through modulation of Bax/Bcl-2 and caspase-3, in some cases surpassing N-acetylcysteine [82,84]. Additional mechanisms include possible chemical sequestration of cisplatin by selenol species and reduction in hyperhomocysteinemia [83], both potentially limiting tubular toxicity. Functional protection is evident in acute models [82,83,84] and in a trial showing reduced RIFLE-defined injury during cisplatin therapy [85], yet it is absent or reversed under repeated cisplatin dosing, particularly with nano-selenium, despite favorable oxidative biomarkers [81], suggesting that benefits depend critically on formulation, dosing, timing and injury context. This divergence underscores that while selenium can attenuate oxidative, inflammatory and apoptotic drivers of nephrotoxicity, chronic co-exposure may foster renal accumulation, redox-cycling or ROS-independent injury, necessitating the careful design of regimens and functional endpoint monitoring [81,82,83,84,85,86].

**Figure 3:**
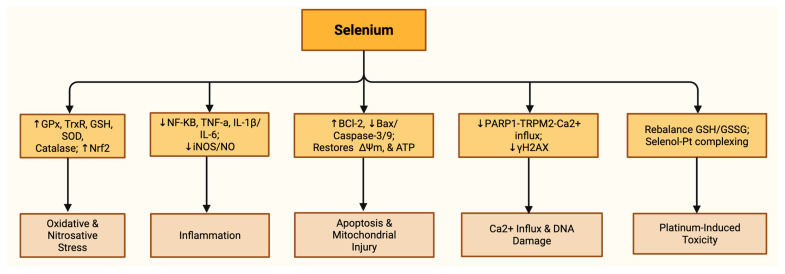
Chemotherapy-induced injury pathways and selenium’s protective mechanisms. Selenium counters these effects by enhancing antioxidant defenses (↑ GPx, TrxR, GSH, SOD, catalase; ↑ Nrf2), suppressing inflammatory mediators (↓ NF-κB, TNF-α, IL-1β/IL-6; ↓ iNOS/NO), promoting anti-apoptotic signaling (↑ Bcl-2, ↓ Bax/caspase-3/9; restoring ΔΨm and ATP), reducing PARP1–TRPM2–Ca^2+^ influx and DNA damage marker γH2AX and mitigating platinum toxicity via GSH/GSSG balance and formation of selenol–Pt complex. Note: ↓: decreased; ↑: increased.

Proposed mechanisms of selenium’s protective role in mitigating chemotherapy-induced organ injury are summarized in Table 4 and Figure 3.

### 4.3. Zinc

#### 4.3.1. Protective Role of Zinc in Chemotherapy-Induced Hepatotoxicity

In rat models of anticancer drug-induced liver injury, namely tamoxifen-associated hepatotoxicity and doxorubicin (DOX)-induced hepatic damage, zinc demonstrated consistent hepatoprotective effects through restoration of hepatic biochemical integrity and protein synthesis, reversal of aminotransferase elevations and attenuation of necrosis, steatosis, hydropic degeneration and inflammatory infiltration, thereby preserving hepatic architecture [87,88]. Central to these effects was the re-establishment of redox homeostasis, with zinc preventing or reversing glutathione depletion, reinstating superoxide dismutase, catalase and glutathione peroxidase activities and reducing malondialdehyde levels, which collectively curtailed ROS propagation and stabilized redox-sensitive proteins [87,88]. Zinc further activated cytoprotective transcriptional programs, upregulating heme oxygenase-1 and UDP-glucuronosyltransferases (UGT1A1/UGT2B1) suppressed by DOX, thus enhancing heme catabolism and conjugative clearance of toxic intermediates [88]. Anti-inflammatory actions included inhibition of NF-κB activation, inducible nitric oxide synthase expression, nitric oxide overproduction and suppression of proinflammatory cytokines (IL-6, IL-1β, TNF-α), mitigating oxidative stress-driven inflammatory cascades [87]. In parallel, zinc reduced apoptosis via caspase-3 inhibition in tamoxifen injury and suppression of pro-apoptotic JNK signaling in DOX toxicity through MAPK phosphatase 1 upregulation, leading to selective JNK dephosphorylation [87,88]. These integrated mechanisms underpin zinc’s capacity to stabilize hepatocyte membranes, restrain oxidative and inflammatory injury, interrupt apoptosis and bolster detoxification, with taurine–zinc solid dispersions exhibiting superior efficacy over taurine alone, physical taurine–zinc mixtures and silymarin, likely reflecting enhanced bioavailability and a substantive zinc-dependent contribution [88].

#### 4.3.2. Protective Role of Zinc in Chemotherapy-Induced Cardiotoxicity

In both cellular and animal anthracycline cardiotoxicity models, zinc supplementation, particularly as Zn(II)–curcumin or taurine–zinc solid dispersions, preserves myocardial structure and function by correcting zinc dyshomeostasis and interrupting oxidative, inflammatory and apoptotic pathways [88,89]. Doxorubicin disrupts systemic and cardiac zinc transport, depresses plasma and myocardial zinc, upregulates ZnT1 and downregulates ZIP5, metallothionein (MT) and intestinal ZIP4; zinc formulations restore these parameters, aligning with normalization of electrocardiographic, hemodynamic and histological indices and preservation of mitochondrial ultrastructure [89]. MT is indispensable for protection, as zinc elevates MT; prevents apoptosis, DNA damage, lipid peroxidation, and ROS accumulation; and maintains antioxidant enzyme and peroxiredoxin function only in MT-competent contexts, effects absent in MT-null settings [90,91]. This MT-dependent antioxidant buffering constrains the formation of peroxynitrite, mitigates mitochondrial and calcium-handling impairment and is reinforced by induction of heme oxygenase-1, phase II detoxification enzymes and MAPK phosphatase 1, leading to suppression of c-Jun N-terminal kinase phosphorylation, reduced caspase-3 and Bax, increased Bcl-2 and downregulation of Egr1 and proinflammatory cytokines [88,89]. Zinc also counteracts doxorubicin-induced gut dysbiosis, barrier disruption, and endotoxemia, restoring short-chain fatty acid-producing taxa, tight junction proteins and mucus integrity, with microbiota changes correlating with zinc bioavailability and myocardial protection and transferable by transplantation of fecal microbiota independent of fecal zinc or curcuminoids [89]. Additionally, zinc modulates daunorubicin–myosin binding in a concentration-dependent manner, with physiologic Zn^2+^/Cu^2+^ balance diminishing binding and thereby supporting contractile integrity [92].

#### 4.3.3. Protective Role of Zinc in Chemotherapy-Induced Nephrotoxicity

Zinc functions as a context-dependent modulator of cisplatin nephrotoxicity, whose efficacy is contingent on metallothionein (MT) competence, redox modulation, species differences, dosing and timing. In MT-competent mice, zinc sulfate pretreatment significantly reduced blood urea nitrogen and plasma creatinine, an effect absent in MT-null counterparts, indicating that MT induction is essential for renoprotection through sequestration of platinum species and suppression of lipid peroxidation and oxidative injury [93]. Complementary work with zinc gluconate demonstrated early attenuation of creatinine and urea elevations, reduced malondialdehyde and partial glutathione restoration despite incomplete recovery of antioxidant enzyme activity, suggesting interruption of peroxidative chain reactions and membrane stabilization [94]. Conversely, in rats, high-dose parenteral zinc failed to mitigate cisplatin-induced azotemia despite marked MT induction, and it was associated with persistent suppression of zinc-dependent enzymes, altered angiotensin-converting enzyme activity, trace-metal imbalances and off-target reproductive toxicity, implying that supra-physiological exposure can disrupt homeostatic networks and negate MT-mediated benefits [95]. These findings imply that protective effects require synchronizing MT and thiol availability with peak nephron vulnerability, and that excessive dosing, inappropriate formulations, or species-specific pharmacodynamics may abrogate their efficacy [93,94,95].

**Figure 4:**
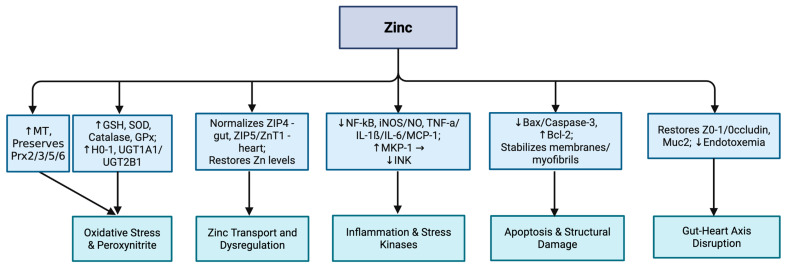
Chemotherapy-induced injury pathways and zinc’s protective mechanisms. Zinc mitigates these pathways by inducing metallothionein and preserving peroxiredoxins, boosting antioxidant and phase II conjugation capacity (GSH, SOD, catalase, GPx; HO-1; UGT1A1/UGT2B1), normalizing intestinal and cardiac zinc transporters (ZIP4, ZIP5, ZnT1) and tissue Zn levels, dampening NF-κB/iNOS–NO and cytokines with MKP-1-mediated restraint of JNK, limiting Bax/caspase-3-dependent apoptosis while supporting Bcl-2, and restoring epithelial barriers (ZO-1, occludin, Muc2) to reduce endotoxemia. Note: ↓: decreased; ↑: increased.

Proposed mechanisms of zinc’s protective role in mitigating chemotherapy-induced organ injury are summarized in Table 5 and Figure 4.

### 4.4. Vitamin D

In the past it has been reported that deficiency/insufficiency of vitamin D can play an important role in causing various pathological conditions, including inflammation, oxidative stress, vascular remodeling and endothelial dysfunction, which are the crucial risk factors for numerous CVDs, including coronary artery disease (CAD), myocardial infarction (MI), pulmonary arterial hypertension (PAH), left ventricular hypertrophy, cardiomyopathy, fibrosis and heart failure (HF) [96]. Additionally, studies also witnessed that vitamin D mediates the promotion of several effects. such as anti-inflammatory, anti-oxidant, anti-fibrotic and anti-hypertensive ones, through regulation of the expression of different signaling pathways [97,98].

#### 4.4.1. Protective Role of Vitamin D in Chemotherapy-Induced Cardiotoxicity

In both preclinical and clinical contexts, activation of the vitamin D–vitamin D receptor (VDR) axis consistently mitigates anthracycline-induced cardiotoxicity through interlinked mitochondrial, inflammatory and electrophysiological mechanisms, with evidence spanning rodent models and human supplementation studies [99,100,101,102]. Mechanistically, vitamin D3 or the VDR agonist paricalcitol preserves mitochondrial integrity by reducing doxorubicin-evoked ROS, restoring membrane potential, sustaining succinate dehydrogenase activity, restraining DRP1-mediated fission and lowering lipid peroxidation, thereby limiting apoptotic signaling via reduced cleaved caspase-3 [99,101,102]. Concurrently, it suppresses pro-inflammatory cytokines (TNF-α, IL-6) and nitric oxide, downregulates iNOS and inhibits NF-κB-dependent transcription, effects correlating with improved cardiac electrophysiology, including normalization of heart rate, QT interval, QRS width and ST-segment changes, alongside reductions in troponin T, lactate dehydrogenase and 99mTc-pyrophosphate uptake [99,100]. In triple-negative breast cancer models, supplementation preserved left ventricular systolic function at cardiotoxic doxorubicin doses without impairing, and in some settings enhancing, antitumor efficacy, while clinical data demonstrated biomarker-level cardioprotection proportionate to achieved serum vitamin D concentrations [100,101]. Collectively, these findings delineate a coherent cascade in which VDR activation stabilizes mitochondrial energetics, restrains oxidative and inflammatory amplification and thereby maintains myocardial structural and functional integrity during anthracycline therapy [99,100,101,102].

#### 4.4.2. Protective Role of Vitamin D in Chemotherapy-Induced Nephrotoxicity

Vitamin D signaling exerts multifaceted renoprotective effects against chemotherapy-induced nephrotoxicity, principally in doxorubicin, cisplatin and adriamycin models, through coordinated modulation of inflammatory, fibrotic, apoptotic and vasoactive pathways [103,104,105]. In both doxorubicin-bearing tumor mice and cisplatin injury, active vitamin D or analogs attenuated NF-κB activation, reduced pro-inflammatory mediators such as MCP-1 and TNF-α and downregulated NF-κB, thereby curbing leukocyte infiltration and cytokine amplification; in silico docking further suggested direct interference with MCP-1 [103,104]. Antifibrotic actions included limiting interstitial collagen, suppressing α-SMA, rebalancing TGF-β/Smad signaling by downregulating TGF-β1 and Smad3 while restoring Smad7 and blunting JNK1 activation, with docking indicating stable JNK1 occupancy by vitamin D [103]. Apoptotic signaling, notably caspase-3 activity, was reduced in cisplatin models alongside these upstream changes [104]. In cisplatin injury, vitamin D also recalibrated the endothelin axis by lowering ET-1 and ETAR, upregulating ETB, and restoring vitamin D receptor expression, thereby enhancing vasodilatory and anti-inflammatory tone; reciprocal VDR–ETAR modulation suggested a positive feedback loop in injury that was disrupted by therapy [104]. Functionally, these mechanisms improved creatinine, urea and histological indices in cisplatin and doxorubicin contexts, ameliorated tubular lesions in adriamycin nephropathy without short-term proteinuria reduction and preserved antitumor efficacy, with combined VDR activation and ETAR blockade showing superior benefit in cisplatin injury [103,104,105].

**Figure 5:**
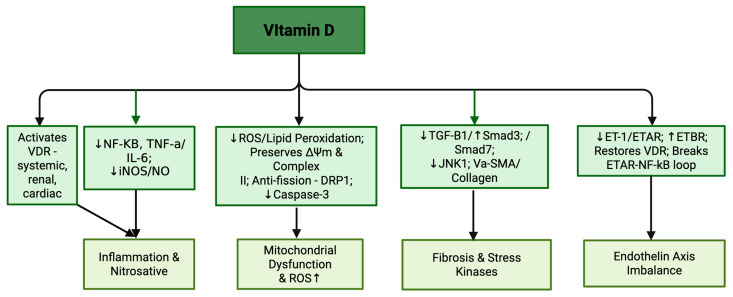
Chemotherapy-induced injury pathways and vitamin D’s protective mechanisms. Vitamin D acts via the VDR in systemic, renal and cardiac tissues to suppress ROS and lipid peroxidation, preserve ΔΨm and complex II activity, inhibit mitochondrial fission (DRP1) and reduce caspase-3 activation. It downregulates NF-κB, TNF-α, IL-6, iNOS and NO, attenuates fibrosis by modulating TGF-β1/Smad3 and Smad7, inhibits JNK1, α-SMA and collagen deposition and restores endothelin axis balance by decreasing ET-1/ETAR, increasing ETBR, re-establishing VDR expression and disrupting the ETAR–NF-κB feedback loop. Note: ↓: decreased; ↑: increased.

Proposed mechanisms of Vitamin D’s protective role in mitigating chemotherapy-induced organ injury are summarized in Table 6 and Figure 5.

### 4.5. Limitations

Several important limitations should be considered when interpreting the findings of this review. Although the preclinical evidence base is extensive, the quantity and quality of clinical trial data remain limited, with available human studies predominantly comprising small, single-center, nonrandomized investigations. Such constraints impede precise estimation of effect sizes and restrict the external validity of conclusions. The status of clinical research reflects a scarcity of rigorously designed randomized controlled trials (RCTs), and advancing this field will require well-powered, multicenter RCTs that incorporate standardized protocols and clearly defined endpoints. Moreover, the existing variability in patient populations, cancer types, chemotherapeutic regimens, micronutrient formulations, dosing schedules, timing of administration and outcome definitions contributes to substantial heterogeneity, necessitating cautious synthesis and interpretation. Furthermore, the narrative approach adopted here was appropriate to capture the breadth and diversity of the evidence. However, it cannot overcome inherent biases in the literature, including publication bias, restriction to English-language studies and time-lag bias. The literature search was completed on 29 June 2025, and thus later-emerging data were not captured. Across the included studies, outcome measures and safety reporting were inconsistent, with a notable lack of harmonized definitions for cardiotoxicity, hepatotoxicity and nephrotoxicity; limited use of validated biomarkers; and infrequent long-term follow-up. There was also insufficient assessment of oncologic outcomes and of pharmacokinetic or pharmacodynamic interactions that might influence anticancer efficacy. Moreover, formulation-specific findings may not be generalizable to more widely available preparations, and several micronutrients possess narrow therapeutic windows, raising the potential for pro-oxidant effects or organ-specific toxicity at higher doses. While dual screening was performed to enhance the rigor of study selection, a formal, study-level risk-of-bias assessment and quantitative meta-analysis were beyond the scope of this review. Collectively, these factors underscore the urgent need for future clinical research, particularly large-scale, methodologically robust RCTs employing standardized outcome measures and mechanistic biomarkers, together with prespecified oncologic safety assessments, to validate and translate these promising preclinical and early-phase clinical signals into safe, effective and evidence-based supportive care strategies.

## 5. Conclusions

The present review delineates the breadth and mechanisms of chemotherapy-related organ toxicities and synthesizes evidence for the adjunctive role of key micronutrients, magnesium, selenium, zinc and vitamin D, in mitigating cardiotoxicity, hepatotoxicity and nephrotoxicity. Across systems, a convergent pathobiology centers on oxidative stress, inflammatory signaling, mitochondrial dysfunction and, in selected contexts, ferroptosis. Within this framework, the evaluated micronutrients consistently reinforced endogenous antioxidant capacity, stabilized mitochondrial function and downregulated pro-inflammatory and pro-apoptotic pathways, yielding coherent biochemical, histological and functional protection in preclinical models, with emerging, though heterogeneous, clinical signals.

Among the micronutrients reviewed, magnesium currently has the clearest clinical utility. Routine intravenous supplementation within cisplatin hydration protocols is supported by multicenter cohort data and meta-analytic evidence showing reduced nephrotoxicity. Selenium shows selective clinical promise (preserved GFR with selenium plus vitamin E during cisplatin therapy and improvement in selenium-deficient pediatric patients on anthracyclines), but its nephroprotective effect is formulation- and schedule-dependent. Vitamin D demonstrates biomarker-level cardioprotection in patients receiving anthracyclines and preserves cardiac function in preclinical models without compromising antitumor efficacy, warranting larger outcome-driven trials. Zinc’s benefits are compelling preclinically yet remain to be validated clinically.

Compared with established agents, dexrazoxane retains primacy for anthracycline cardioprotection, while phytotherapeutics such as silymarin, N-acetylcysteine and curcumin have broader real-world use in hepatobiliary support. Mechanistically, selenium and zinc offer antioxidant and anti-inflammatory effects comparable to these agents, with selenium showing some clinical preservation of renal function when paired with vitamin E. However, real-world use is more established for silymarin and N-acetylcysteine in hepatobiliary support, whereas selenium’s renal benefit is formulation-sensitive, and zinc’s role remains preclinical. The micronutrients evaluated in this review appear best positioned as adjuncts: magnesium as standard in cisplatin protocols, selenium and vitamin D as targeted options pending regimen-specific validation and zinc as investigational, guided by metallothionein biology.

Translationally, these findings support the rational incorporation of micronutrients into supportive oncology care. However, the preponderance of animal data and variability in human study design, dosing and outcomes warrant caution. Priorities for future research include rigorously powered randomized clinical trials that stratify by cancer type, regimen and baseline micronutrient status; dose–response and formulation studies to define therapeutic windows and avoid pro-oxidant toxicity; and integration of mechanistic biomarkers, encompassing mitochondrial function, redox indices and immune signaling, to enable response monitoring and patient selection. Equally critical is the prospective evaluation of oncologic safety to exclude attenuation of chemotherapy efficacy and to clarify potential synergistic or antagonistic interactions.

Concluding, the available evidence positions magnesium, selenium, zinc and vitamin D as biologically plausible, potentially cost-effective adjuncts for reducing chemotherapy-related organ toxicities. Confirmatory clinical trials with standardized endpoints and mechanistic correlative studies are needed to translate these promising signals into evidence-based protocols, optimize patient-centered dosing strategies and ultimately enhance the tolerability and continuity of cancer therapy.

## Figures and Tables

**Table 1:** Chemotherapy-induced toxicity per system. Note: ↓: decreased.

Organ-Specific Toxicity	Common Chemotherapy Agents Involved	Mechanisms of Toxicity	Clinical Manifestations	Diagnostic Monitoring Notes
Cardiotoxicity [17,18,19,20,21,22]	Anthracyclines (doxorubicin), trastuzumab, cyclophosphamide, ifosfamide, cisplatin, paclitaxel, etoposide, vinca alkaloids, fluorouracil, cytarabine, etc.	Free radical generation, oxidative stress, myofibrillar disarray (Type I), transient cardiomyocyte dysfunction (Type II), possible overlap in pathogenesis	HF symptoms, ↓ LVEF, arrhythmias, myocardial ischemia, hypertension; acute (<1%), chronic (1.6–5%), asymptomatic LVEF fall, biomarker rises	Defined by LVEF criteria; Type I often permanent, dose-dependent; Type II usually reversible; MRI may show scarring even in Type II
Neurotoxicity [23,24,25]	Vincristine, methotrexate, cisplatin, cytarabine, brentuximab vedotin, blinatumomab	Direct neurotoxicity, oxidative stress, mitochondrial dysfunction, barrier disruption	Peripheral neuropathy (sensory loss, paresthesia), seizures, encephalopathy, delirium, cerebellar dysfunction	Neurotoxicity is 2nd most common dose-limiting factor after myelosuppression; barriers offer partial protection
Nephrotoxicity [26,27,28,29,30,31]	Cisplatin, methotrexate, gemcitabine, EGFR inhibitors, immune checkpoint inhibitors	Direct tubular injury, oxidative stress, thrombotic microangiopathy; exacerbated by dehydration, other nephrotoxins, preexisting CKD	AKI, CKD, proteinuria, electrolyte imbalances	Early risk identification essential; preventive strategies (hydration, dose adjustment) important
Pulmonary Toxicity [32,33]	Anthracyclines, various antitumor antibiotics; doxorubicin noted in angiosarcoma lung fibrosis	ROS generation, pneumocyte/endothelial damage, hypersensitivity reactions (Gell & Coombs I–IV)	Fever, cough, dyspnea; patterns include interstitial pneumonia, alveolar damage, eosinophilic pneumonia, pulmonary hemorrhage	High-resolution CT preferred for evaluation; often compounded by opportunistic infections
Gastrointestinal Toxicity [34]	Irinotecan, 5-FU, cytarabine, cisplatin, taxanes	Epithelial barrier injury, inflammation, dysbiosis, oxidative stress, altered ion channels and neuronal excitability	Diarrhea (up to 80%), mucositis, malabsorption, villus atrophy	Involves 5-phase mucositis pathobiology; gut microbiome crucial; current treatments mostly palliative
Hepatotoxicity [35,36,37,38,39,40]	Methotrexate, cyclophosphamide, oxaliplatin, irinotecan, various others	Oxidative stress, mitochondrial dysfunction, immune-mediated inflammation	Transaminase elevations to acute liver failure; steatohepatitis, sinusoidal obstruction syndrome	Often idiosyncratic; higher risk in preexisting liver disease/viral hepatitis; monitor LFTs during therapy

**Table 2:** Summary of common chemotherapy-induced toxicities, the potential protective micronutrients and the proposed mechanism of protection [13,14,15,16,17,18,19,20,21,22,23,24,25,26,27,28,29,30,31,32,33,34,35,36,37,38,39,40,41,42,43,44,45,46].

Toxicity	Protective Agents	Mechanism of Action	Comments
Cardiotoxicity	− Dexrazoxane (1000 mg/m^2^) − Coenzyme Q10 (200 mg/day) − Selenium (100–200 μg/day) − Vitamin E (300 or 600 mg/day) − Omega-3 fatty acids (500 mg/day) − Berberine (10–20 mg/kg/day)	− Iron chelation, reduces ROS − Supports mitochondrial function − Antioxidant defense − Anti-inflammatory	Dexrazoxane approved for anthracycline-induced cardiotoxicity
Neurotoxicity	− Magnesium (250 mg/day) − Vitamin B6 (up to 100 mg/day) − Vitamin B12 (100 µg/day) − Alpha-lipoic acid (35 mg/kg/week) − >Glutamine (500–600 mg/kg/day)	− Stabilizes neuronal membranes − Supports myelin synthesis − Antioxidant, improves mitochondrial function − Reduces excitotoxicity	Effective in oxaliplatin and vincristine-induced neuropathy
Nephrotoxicity	− Magnesium (250 mg/day) − N-acetylcysteine (600 mg/day) − Selenium (100–200 μg/day)	− Dilutes nephrotoxins − Prevents electrolyte imbalance − Boosts glutathione − Reduces tubular oxidative damage	Critical for cisplatin and methotrexate protocols
Pulmonary Toxicity	− N-acetylcysteine (600 mg/day) − Vitamin C (1 g/day) − Vitamin E (300 or 600 mg/day) − Curcumin (50–100 mg/kg/day)	− Antioxidant and mucolytic − Reduces inflammation − Scavenges ROS − Modulates cytokine response	Used in drug-induced pneumonitis and interstitial lung disease
Gastrointestinal Toxicity	− L-glutamine (500–600 mg/kg/day) − L-Cystine (up to 2100 mg/day) − L-Theanine (up to 840 mg/day) − Vitamin D (1000 IU/day) − Probiotics (e.g., 250–500 mg/day for *S. boulardii*)	− Supports mucosal repair − Reduces ROS and apoptosis − Modulates immune response − Restores microbiome balance	Promising in mucositis and chemotherapy-induced diarrhea
Hepatotoxicity	− Silymarin (milk thistle; 60 mg/kg/day) − N-acetylcysteine (600 mg/day) − Vitamin E (300 or 600 mg/day) − Ursodeoxycholic acid (up to 20 mg/kg/day)	− Antioxidant and hepatoprotective − Enhances glutathione synthesis − Reduces lipid peroxidation − Improves bile flow and reduces cholestasis	Especially relevant for methotrexate, irinotecan and platinum agents

**Table 3:** Summary of the protective role of magnesium in mitigating renal, liver and cardiac chemotherapy-induced toxicity. Note: ↓: decreased; ↑: increased.

Model or Population	Magnesium Form and Regimen Used	Key Outcomes	Proposed Mechanisms and Notes
Nephrotoxicity			
Adults receiving first-dose cisplatin (multicenter cohort) [56]	Intravenous magnesium (median 2 g on day of chemotherapy)	Lower AKI or death within 2 weeks (OR 0.80; 95% CI 0.66–0.97); signals of greater benefit in <65 years, women, diabetes, baseline eGFR ≥ 90, and baseline Mg 2.0–2.2 mg/dL; protection persisted to MAKE90; no excess adverse events reported	Mg repletion likely normalizes proximal tubular platinum handling (↓ OCT2-mediated uptake, ↑ MATE1 efflux), stabilizes membranes, supports Na^+^/K^+^-ATPase, modulates Ca^2+^ channels and dampens oxidative/inflammatory cascades; fixed 1–2 g dosing may be suboptimal for some patients, inviting baseline- or weight-guided strategies and timing within hydration protocols
Meta-analysis of adult studies (11 retrospective studies; n = 1688) [57]	Magnesium co-administration with cisplatin (varied protocols)	Markedly lower risk of cisplatin nephrotoxicity (pooled OR 0.22; 95% CI 0.14–0.35), strongest at cisplatin ≤ 50 mg/m^2^ but benefit across dosing strata	Supports routine Mg in cisplatin hydration protocols; effect sizes suggest clinically meaningful protection across regimens
Pediatric oncology, carboplatin (randomized trial) [58]	Oral magnesium oxide 250 mg/day for 2 weeks	No reduction in early nephrotoxicity biomarkers; similar changes in creatinine, BUN and eGFR between arms	Boundary condition: short-course, low-dose oral Mg during carboplatin did not show benefit; findings should not be over-generalized to cisplatin or to IV Mg strategies
**Hepatotoxicity**			
Alectinib-induced liver injury (mouse model) [59]	MgIG 25 mg/kg/day	Biochemical, molecular and histologic hepatoprotection; restored mitochondrial potential and ETC activity; reduced pyroptosis markers (NLRP3, caspase-1 p20, GSDMD-N) and IL-1β	Mitochondria-first protection: MgIG limits ROS, normalizes Nrf2/HO-1 and NF-κB p65, and blocks mitochondrial damage-mediated inflammasome/pyroptosis; NAC parallels underscore ROS centrality
Methotrexate liver–gut axis (mouse model) [60]	MgIG 40 mg/kg/day	Improved ALT/AST, cytokines, oxidative indices and histology; restored tight junctions (ZO-1, claudin-1), ↓ permeability and LPS, ↓ hepatic TLR4, immune cell infiltration; shifted macrophages to M2; FMT and Lactobacillus recapitulated benefits	Gut–liver axis: barrier restoration and microbiota remodeling drive hepatic anti-inflammatory effects; consistent with low systemic bioavailability and biliary/intestinal actions
Arsenic trioxide (ATO) acute liver injury (mouse model) [61]	MgIG 50 and 25 mg/kg/day	↓ ROS/MDA; ↑ SOD, catalase; ↓ IL-1β, IL-6, TNF-α; anti-apoptotic reprogramming (↓ Bax, caspase-3; ↑ Bcl-2); improved histology	Rebalancing Keap1–Nrf2 (↓ Keap1, ↑ Nrf2) aligns transcriptional cytoprotection with antioxidant recovery
Methotrexate hepatointestinal injury (rat model) [62]	MgIG 9 and 18 mg/kg/day	↓ MDA; ↑ GSH, SOD, GPx; ↓ TUNEL, cleaved caspase-3, Bax, cleaved PARP; ↑ Bcl-2; antifibrotic (↓ collagen I, Sirius red); ↓ COX-2; ameliorated diarrhea and hyperpermeability; restored ZO-1	Dual hepatic–intestinal anti-inflammatory and antifibrotic actions; COX-2 suppression may bridge gut and liver benefits; biliary excretion supports local exposure
**Cardiotoxicity**			
Arsenic trioxide cardiotoxicity (rodent model) [63]	MgIG 50 and 25 mg/kg/day	Restored antioxidant enzymes (SOD, catalase, GPx); ↓ ROS/MDA and pro-inflammatory cytokines; downregulated TLR4/NF-κB	Nrf2 activation with concurrent TLR4/NF-κB suppression interrupts oxidative–inflammatory feed-forward injury
Doxorubicin acute cardiotoxicity (rat models) [64]	MgIG 120 mg/kg/day	↓ Lipid peroxidation; ↑ antioxidant enzymes; anti-apoptotic shift (↓ Bax, caspase-3; ↑ Bcl-2); ↓ NF-κB p65; lower CK/CK-MB/LDH; histologic and functional preservation	Integrates redox containment, inflammatory control and mitochondrial apoptosis restraint into organ-level protection
Doxorubicin cardiotoxicity (rat model) [65]	MgIG 10 or 20 or 40 mg/kg/day	Corrected QT prolongation; restored papillary muscle excitability and contractile force; replenished cardiac GSH without raising serum Mg	Likely Ca^2+^ antagonism (↓ L-type current), support of SR Ca^2+^ cycling, moderated Na^+^/Ca^2+^ exchange and ↑ Na^+^/K^+^-ATPase stabilize electrophysiology and contraction
Cyclosporine A cardiotoxicity (rat model) [66]	Dietary magnesium (±potassium)	Attenuated coronary luminal narrowing, intima–media thickening, perivascular fibrosis, LV scarring; prevented systolic BP rise	Magnesium repletion mitigates salt-sensitive vasoconstriction/endothelial dysfunction and microvascular remodeling, reducing hemodynamic stress on myocardium

**Table 4:** Summary of the protective role of selenium in mitigating renal, liver and cardiac chemotherapy-induced toxicity.

Model or Population	Regimen	Key Outcomes	Proposed Mechanisms and Notes
Hepatotoxicity			
Mice, doxorubicin-induced liver injury [69]	Turmeric extract–loaded selenium nanoparticles (Tur-SeNPs)	Lowered lipid peroxidation and nitric oxide; restored SOD, catalase, GPx, GR, GSH; improved transaminases/bilirubin; preserved histology	Activated Nrf2; downregulated NF-κB p65, IL-1β, TNF-α, iNOS; reduced apoptosis; broad antioxidant and anti-inflammatory effects
Rats, doxorubicin-induced liver injury [70]	Selenium (low dose 0.5–1 mg/kg protective; 2 mg/kg harmful)	Preserved architecture; limited fibrosis; normalized glycogen; suppressed TNF-α, IL-1β, PCNA	U-shaped dose–response; decreased MDA, increased SOD; GSH-Px/catalase not consistently restored
Rats, adriamycin-induced hepatic mitochondrial dysfunction [71]	Sodium selenite 50 µg/kg/day	Reduced mitochondrial/cytosolic oxidant status; restored mitochondrial membrane potential; increased hepatic mitochondrial ATP; elevated TAS	Recovery of oxidative phosphorylation; augmentation of antioxidant capacity beyond baseline
Mice, cyclophosphamide injury [72]	Selenium nanoparticles (Nano-Se); oral at 2 mg/kg/day	Lower ROS/lipid peroxidation in liver and bone marrow; restored GSH, GPx, GST, SOD, catalase; normalized transaminases; preserved histology; reduced DNA strand breaks	Pre-emptive redox fortification; enhanced conjugative detoxification (GST) leading to lower genotoxic burden
Rats, cisplatin-induced liver injury [73]	Selenium pretreatment at 6 mg/kg/day	Lowered lipid peroxidation; restored GSH, lowered GSSG; normalized GSH/GSSG ratio	Rebalanced glutathione redox; likely enhanced selenoenzymes (GPx, TrxR); potential selenol–cisplatin complexation in normal tissue
Rats, doxorubicin-induced liver injury [74]	Selenium 15 mg/kg/day	Decreased MDA; robust SOD elevation; limited changes in GSH-Px/catalase	Benefit primarily via superoxide handling (SOD) upstream of peroxidase activity
**Cardiotoxicity**			
Rats, doxorubicin-induced cardiotoxicity [75]	Selenium 1 mg/kg/day	Reduced cardiac injury biomarkers; preserved myocardial architecture	Restored redox homeostasis (GSH, SOD, GPx); reduced lipid peroxidation; anti-apoptotic effects
Rats, doxorubicin model [76]	Selenium 0.5 mg/kg/day	Lowered ROS and PARP1; reduced TRPM2 expression; decreased caspase-3	Modulated PARP1–TRPM2–Ca^2+^ axis; upstream antioxidation limiting Ca^2+^-dependent apoptosis
Rats, cyclophosphamide-induced cardiotoxicity [77]	Selenium 1 mg/kg/day	Reduced γH2AX; corrected Bax/Bcl-2; preserved cardiac troponin I; histologic protection	Anti-apoptotic shift; mitigation of DNA damage; membrane/structural stabilization
Mice, doxorubicin-induced cardiomyopathy [78]	Selenium-enriched diet	Preserved ejection fraction; lowered circulating injury markers	Required Nrf2 activation; dampened inflammatory mediators (e.g., TNF-α, IL-1β/IL-18, ICAM-1); Nrf2 inhibition abrogated benefit
Rats, cyclophosphamide-induced cardiotoxicity [79]	Selenium 0.5 and 1 mg/kg/day	Lower ischemia-modified albumin and injury enzymes; improved endothelial/microvascular integrity	Antioxidant and anti-inflammatory effects; vascular protection complements myocardial rescue
Children receiving anthracyclines [80]	Selenium 100 μg/day supplementation in deficient patients	Reduced ProBNP; echocardiographic improvements in several cases	Selenium deficiency clustered with cardiotoxicity; repletion associated with biomarker and functional gains (observational)
**Nephrotoxicity**			
Rats, repeated-cycle cisplatin [81]	Sodium selenite 0.1 mg/kg/day and nano-selenium 0.1 mg/kg/day; prolonged co-exposure	Worsened creatinine/urea; greater structural injury despite improved antioxidant readouts	Formulation- and schedule-dependent harm; antioxidant markers not reliable surrogates for protection under chronic co-dosing
Mice, acute cisplatin nephrotoxicity [82]	Turmeric–selenium nanoparticles 0.5 mg/kg/day	Normalized creatinine/urea; lowered KIM-1/NGAL; ameliorated tubular/glomerular lesions	Suppressed NF-κB, TNF-α, IL-6; activated Nrf2–HO-1; shifted Bax/Bcl-2 toward survival; efficacy comparable/superior to NAC in select endpoints
Rats, cisplatin (post-injury treatment) [83]	Sodium selenite 0.5 mg/kg/day; post-injury dosing	Partial correction of azotemia; improved histology; no harm in healthy rats	Lowered IL-1β, TNF-α, MMP-9; normalized ceramide; reduced homocysteine (candidate mediator/biomarker)
Rats, cyclophosphamide-induced kidney injury [84]	Selenium 0.5 or 1 mg/kg/day; dose-dependent	Improved redox indices; modest functional improvement; preserved renal architecture	Antioxidant restoration driving structural protection
Cancer patients on cisplatin-based chemotherapy [85]	Selenium 200 μg/day plus vitamin E 400 IU/day vs. placebo; randomized	Higher GFR across cycles; fewer RIFLE-defined injuries; benefit persisted 1 month; chemotherapy continued	Clinical preservation of renal function with adjunct antioxidants; supports translational potential
Rats, cisplatin-induced renal oxidative injury [86]	Selenium 6 mg/kg/day	Reduced lipid peroxidation; improved antioxidant defenses in kidneys	Selenium-supported GPx/antioxidant systems limit cisplatin-driven oxidative damage

**Table 5:** Summary of the protective role of zinc in mitigating renal, liver and cardiac chemotherapy-induced toxicity. Note: ↓: decreased; ↑: increased.

Model or Population	Regimen	Key Outcomes	Proposed Mechanisms and Notes
Hepatotoxicity			
Female rats, tamoxifen-induced liver injury [87]	Zinc 100 mg/kg/day	Normalized aminotransferases; restored albumin/total protein; histologic rescue of necrosis, fatty change, hydropic degeneration and inflammation	Antioxidant restoration (↑ GSH, SOD, CAT, GPx; ↓ MDA); inhibition of NO/iNOS/NF-κB; reduced caspase-3-dependent apoptosis
**Cardiotoxicity**			
Rats, doxorubicin-induced cardiomyopathy [89]	Zn(II)–curcumin 25, 50 and 100 mg/kg/day (dose-responsive)	Preserved ECG/hemodynamics; reduced circulating injury biomarkers; mitigated edema, vacuolization, necrosis, perivascular fibrosis; protected mitochondrial ultrastructure	Restored systemic/myocardial zinc and normalized transporters (ZIP4, ZIP5, ZnT1); dampened IFN-γ/TNF-α/IL-1β/IL-6/MCP-1 and Egr1; corrected gut dysbiosis, tight junctions, endotoxemia; gut–heart–zinc axis highlighted
Cardiomyocytes and mouse hearts, doxorubicin exposure [90,91]	Zinc 300 μmol/kg/day (MT-competent vs. MT-null)	Reduced apoptosis, DNA damage, lipid peroxidation, ROS; preserved peroxiredoxins; cardioprotection absent in MT-null	Metallothionein-dependent protection; MT overexpression required; limits superoxide/nitrosative damage (↓ 3-nitrotyrosine), maintains Prx2/3/5/6
In vitro protein interaction, daunorubicin–cardiac myosin [92]	Physiologic Zn^2+^/Cu^2+^ balance	Reduced daunorubicin–myosin binding at physiologic trace metal levels	Suggests trace metal homeostasis can stabilize myofibrillar function; supraphysiologic Zn^2+^ may have opposite effects
**Cardiotoxicity and Hepatotoxicity**			
Rats, doxorubicin-induced injury [88]	Taurine–zinc solid dispersion 40 or 80 mg/kg/day (outperformed taurine, physical mix, silymarin)	Attenuated cardiac and hepatic toxicity (enzymes and histology) versus comparators	Induced HO-1 and UGT1A1/UGT2B1; ↑ MKP1, ↓ JNK activation; anti-inflammatory/anti-apoptotic shifts (↓ caspase-3, ↓ Bax, ↑ Bcl-2)
**Nephrotoxicity**			
Mice, cisplatin [93]	Zinc sulfate 100 μmol/kg/day; MT-null vs. wild-type	↓ BUN/creatinine in wild-type; no benefit in MT-null	Renoprotection requires metallothionein; MT likely sequesters platinum and limits lipid peroxidation
Mice, cisplatin [94]	Zinc gluconate 100 or 140 or 180 mg/kg/day (benefit plateau 100–180 mg/kg)	Lowered early creatinine/urea; ↓ blood/renal MDA; partial restoration of circulating GSH	Interrupts lipid peroxidation and stabilizes membranes despite incomplete enzymatic recovery; MT/thiol preservation implicated
Rats, cisplatin [95]	Zinc 90 mg/kg/day IP	No improvement in urea/creatinine despite ↑ serum Zn and renal MT; enzyme perturbations; ACE increased with zinc alone; trace-metal imbalance	Supra-physiologic zinc may disrupt metal-dependent networks and impose off-target toxicity; MT induction alone insufficient under this context

**Table 6:** Summary of the protective role of vitamin D in mitigating renal, liver and cardiac chemotherapy-induced toxicity.

Model or Population	Regimen	Key Outcomes	Proposed Mechanisms and Notes
Cardiotoxicity			
Rats with acute doxorubicin exposure [99]	Vitamin D3 5000 IU/kg/day	Improved ECG parameters (bradycardia, QT, QRS, ST), reduced cardiac troponin T, decreased 99mTc-pyrophosphate uptake, lower IL-6 and NO	VDR activation dampened NF-κB/iNOS signaling, stabilizing membranes and electrophysiology
Breast cancer patients receiving adjuvant anthracycline–cyclophosphamide [100]	Daily vitamin D (0.5 µg/day) during chemotherapy	Blunted rises in troponin T and LDH; reduced IL-6; inverse correlation between vitamin D increase and biomarker changes	Systemic anti-inflammatory and cardioprotective signaling via VDR; biomarker-level benefit during standard cycles
Triple-negative breast cancer mice on doxorubicin [101]	Dietary vitamin D (1500 IU/kg/day) raising 25(OH)D ~2×	Preserved EF/FS/SV; reduced oxidative lipid peroxidation; decreased cleaved caspase-3	Mitochondrial preservation (stabilized membrane potential; DRP1 phosphorylation shifted anti-fission); antitumor efficacy preserved
Doxorubicin + celecoxib heart failure rat model [102]	Single high-dose vitamin D bolus (60,000 U/kg/day)	Restored mitochondrial potential and complex II (SDH) activity; lowered ROS/MDA; replenished GSH; improved survival	Mitochondria-centric rescue under compounded stress; antioxidant defense enhancement
**Nephrotoxicity**			
Doxorubicin in tumor-bearing mice [103]	Vitamin D 0.5 μg/kg/day IP	Lower serum creatinine/BUN; reduced kidney weight; ameliorated interstitial collagen and cellular infiltration	Repressed NF-κB and MCP-1; antifibrotic TGF-β/Smad3 restraint with Smad7 restoration; JNK1 suppression; in silico MCP-1 binding; antitumor effect maintained
Cisplatin nephrotoxicity in rats [104]	Alfacalcidol (active VDR agonist; 50 ng/kg/day), with/without ETAR blockade	Improved creatinine/urea and histology at 96 h and 14 d; greater benefit with ETAR blockade	Restored VDR; decreased ET-1/ETAR; increased ETBR (NO/prostacyclin tone); suppressed pSer536-NF-κB, TNF-α, TGF-β1; breaks VDR–endothelin inflammatory loop
Adriamycin nephropathy in rats (3-week model) [105]	Cholecalciferol (vitamin D3; 200 IU/day)	Improved tubulointerstitial lesions; proteinuria unchanged	Early tubulocentric cytoprotection without short-term glomerular effect; suggests ligand–timing dependence and need for active analogs or longer duration

## Data Availability

No new data were created or analyzed in this study.

## Supplementary Materials

The following supporting information can be downloaded at: https://www.mdpi.com/article/10.3390/nu17172838/s1, Figure S1: PRISMA flow diagram.
