# Supplementary figures and images for: Protective Role of Key Micronutrients in Chemotherapy-Induced Organ Toxicity: A Comprehensive Review of Mechanistic Insights and Clinical Implications
Source: Nutrients. 2025 Aug 31;17(17):2838. doi: 10.3390/nu17172838 (PMC12430086; doi:10.3390/nu17172838)

Supplementary Figure S1. PRISMA flow diagram.

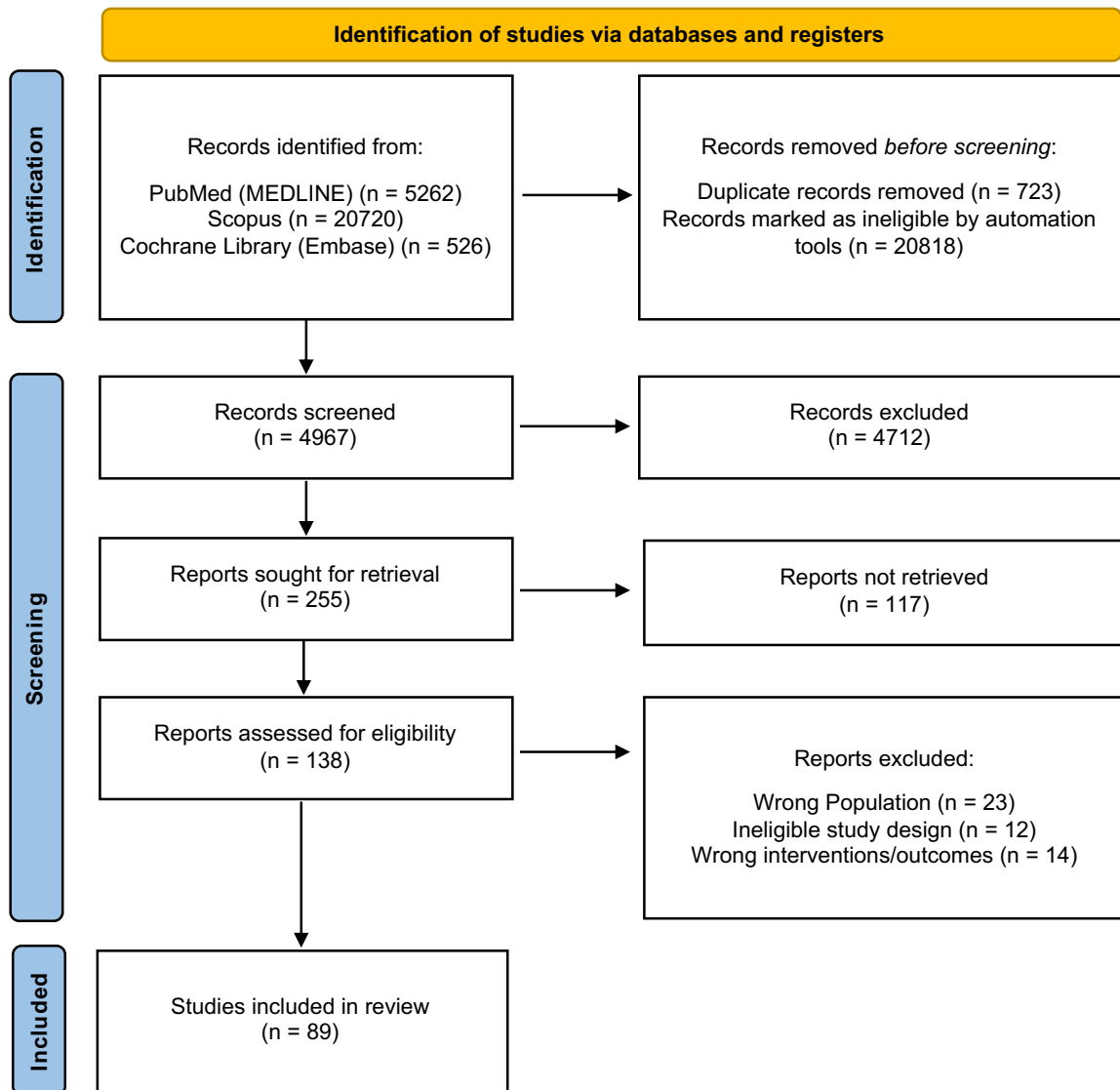

Supplement: Supplementary file 1 [file nutrients-17-02838-s001.zip › nutrients-3840961-supplementary.pdf]
